# Assessing the accuracy of routing engines in replicating children’s walking routes to school: a comparative study of google, mapbox, and OSRM

**DOI:** 10.1186/s12942-026-00473-7

**Published:** 2026-05-19

**Authors:** Hyesop Shin, Jonathan R Olsen, Paul Mccrorie, Fiona Caryl, Melody Smith, Rich Mitchell

**Affiliations:** 1 School of Environment, University of Auckland, Auckland, New Zealand; 2 School of Health and Wellbeing, University of Glasgow, Glasgow, UK; 3 School of Nursing, University of Auckland, Auckland, New Zealand; 4 Institute for Social Science Research, The University of Queensland, Brisbane, Australia; 5 School of Public Health and Sports Sciences, University of Exeter, Exeter, UK

**Keywords:** Active school travel (AST), Routing engines, Overlapping accuracy (OA), Children, GPS, Physical activity

## Abstract

**Background:**

Active school travel (AST) can significantly increase children’s physical activity. AST research often utilises global positioning system (GPS) devices to objectively track the routes children take to school. However, GPS-based studies are costly and often subject to recruitment bias which limits population representativeness. Routing engines, such as Google Maps, designed to help people navigate might be low-cost alternatives to the use of GPS if the engines were able to accurately model the routes children took in AST. To date, no studies have systematically evaluated whether routing engines can serve as a viable alternative for modelling children’s walking routes to school. We assessed whether widely available routing engines can approximate children’s walked routes, providing an internationally transferable, low-cost alternative.

**Methods:**

We compared three routing engines: Google, Mapbox, and Open Source Routing Machine (OSRM), in replicating the GPS measured walking trajectories of 233 school children (age 10–11) across urban and rural Scotland. The percentage of overlap (overlap accuracy, OA), between routing engine predictions and actual GPS tracks was assessed.

**Results:**

Mean OA was 67.6% for OSRM, 66.8% for Mapbox, and 62.6% for Google, indicating that approximately two-thirds of each predicted route overlapped with the GPS-measured track. These values were substantially higher than a shortest-path baseline computed on the Ordnance Survey (OS) road network (45.0%). No significant differences in OA were found by sex, deprivation, or urban/rural setting. However, route distance was negatively associated with OA (*r* = − 0.16 to − 0.23, *p* < 0.05). At an individual level, one-third of participants showed consistent results across all engines, whilst 49.4% had one discordant engine and 17.2% had mutually inconsistent engines.

**Conclusions:**

Contemporary routing engines reproduce roughly two-thirds of children’s walked routes to school, without systematic differences by common sociodemographic or contextual factors. Overall, contemporary routing engines can complement GPS in children’s mobility research and practice, offering an empirical reference point for future AST studies.

**Trial registration:**

Not applicable.

**Supplementary Information:**

The online version contains supplementary material available at 10.1186/s12942-026-00473-7.

## Introduction

Active school travel (AST), which includes walking, wheeling (wheelchairing), cycling, or scooting to and from school, has been linked to numerous benefits for children’s physical and mental health [1–4]. AST also reduces motorised travel dependence, which contributes to lower air and noise pollution, thus benefiting public health and sustainability [5, 6]. Reduced motorised travel in neighbourhoods also reduces the risk of traffic-related deaths and serious injuries for pedestrians and cyclists [7].

Despite the clear health and environmental advantages of AST, its adoption remains low among children in many countries [8]. For instance, only 43% of school children in England in 2022 typically walked or wheeled to school [9], and this figure is even lower in the USA, reaching 11% in 2022 [10]. Data from Scotland suggests recent decline in AST rates from 51.2% in 2020 to 46.6% by 2023 [11].

A myriad of factors impact AST and these operate across individual, family, community, school, neighbourhood environment, and policy levels [12–14]. At the individual level, age and gender play key roles in AST prevalence. While younger children are more likely to be driven to school, they gain independence to walk or wheel to school as they age [15, 16]. Gender differences in travel behaviour become apparent in later childhood, rather than the early years of school [17]. For instance, in New Zealand [18], 6-year-old boys had slightly lower rates of active school transport (AST) than girls, though this difference was not statistically significant. However, when examining changes in transport behaviour during the early school years, boys were twice as likely as girls to switch from passive transport modes to AST between ages 6–8 [18]. When gender differences in routes to school are examined, girls often travel along routes perceived to be safer, even if they are longer [19], whereas boys tend to use shorter routes that may include more high-traffic areas [20].

Socioeconomic position (SEP), either at the individual or area level, also plays an important part in children’s AST. A link between higher deprivation and increased AST has been identified, though findings are inconsistent and vary by location. For example, in Norfolk, England children living in more deprived, higher density areas were more likely to walk to school [21]. A nationwide study in Scotland using travel diaries and national travel survey data also found greater active travel in more deprived areas [22]. However, a meta-analysis in New Zealand found that lower school socioeconomic status was negatively associated with active school travel mode [12], while another New Zealand study found that AST did not differ based on socio-economic deprivation [23].

Further complexity is added by built environment and context. Studies examining the walkability infrastructure around Scottish primary schools found that areas of higher deprivation actually showed better walkability scores compared to more affluent areas [24]. The urban/rural setting presents another critical dimension. Urban areas typically provide better pavements (i.e., sidewalks) and structured pathways (e.g., designated walkways in parks and reserves) which enhance safety and feasibility for AST [25, 26]. By contrast, research focused on rural areas has consistently reported poor or inadequate infrastructure to support children’s AST [27–29].

Understanding children’s AST and key influences such as age, gender, SEP, and urban/rural setting, is imperative for the promotion of safer and healthier travel behaviours. One of the tools most used for measuring children’s routes taken to school in real time is Global Positioning System (GPS) technology [30, 31]. However, GPS data collection can be costly, labour-intensive, burdensome for participants, and ethically challenging due to privacy concerns. These issues make it difficult to implement GPS tracking in population-level studies. Recruitment and adherence biases, common in GPS studies, are also problematic and create challenges for both representativeness and statistical inference, as well as for studies attempting to integrate GPS-based results from small samples with nationwide or targeted population surveys [19, 29, 32].

To address these challenges, some studies have incorporated GIS-based routing engines, to supplement GPS data [29, 32, 33]. These engines use shortest-path algorithms to generate potential routes that children might take to school. Despite their convenience, routing engines have limitations. Studies have shown that children’s journeys to school often do not follow the shortest path due to preferences for avoiding busy junctions, traffic lights, or multiple turns [33–36]. Harrison et al. [35] reported that the distance children actually travelled to school measured using GPS was 6.1–23.2% greater than that suggested or predicted by GIS-based models, and that these differences were driven by the complexity of the built environment between origins and destinations. Although many routing engines tend to default to the shortest path, some GIS routing tools do not draw on networks that identify pedestrian paths or trails, or rely on static pedestrian datasets [35, 37]. The existing studies are also characterised by inconsistent measurement of differences between modelled and GPS-based routes. Studies have used varying buffer distances around modelled routes to determine whether GPS tracks matched the predicted paths, with buffer parameters ranging from 20 m [38] to 25 m [33, 34, 39], 50 m [35, 37], 80 m [40], and 100 m [39].

Recent advancements in mapping techniques, such as those provided by Mapbox and Google Maps that are accessible through application programming interfaces (APIs), offer significant benefits compared to traditional GIS-based routing engines and desktop mapping software [41–43]. These platforms offer rich, continuously updated databases, able to account for relevant factors such as motorised traffic speed, traffic volume (i.e., severe, heavy, moderate, and low), and topographic slope. Whilst these tools may offer useful route suggestions, children’s actual routes are likely shaped by local knowledge, personal preferences, and familiarity with their neighbourhood, and may not closely follow routing engine predictions. It is also unknown whether the accuracy of these models varies by individual or environmental characteristics. Without validation, reliance on GIS routing systems in research could introduce systematic biases. Given variation in routing engine method and data, it is necessary to test several tools to compare performance comprehensively.

Hence, this research aimed to: compare routes children actually took to school, as measured by GPS trajectories, with routes modelled by three routing engines as well as a road-network shortest-path baseline; examine whether accuracy varied by children’s socioeconomic and sex characteristics or urban/rural context; and investigate whether the routing engines were equally accurate for each individual child.

## Data and methods

This section describes the data obtained for this study, the procedure for filtering school route data from the raw GPS data, calculating the overlap accuracy between GPS trajectories and modelled results from routing engines, and the statistical methods used for aggregated and individual level analyses.

### Study population: SPACES dataset

This was a secondary data analysis with participants drawn from the Studying Physical Activity in Children’s Environments (SPACES) study.

The original SPACES participants were selected from Growing up in Scotland (GUS), a comprehensive longitudinal cohort study launched in 2005 (https://growingupinscotland.org.uk/*).* During GUS interviews from September 2014 to February 2015, 2,404 children aged 10–11 years were invited to participate in SPACES, with 2,162 parents providing consent. The SPACES study collected GPS and accelerometer data from children aged 10–11 years between May 2015 and May 2016 [44, 45]. Participants wore GPS devices (Qstarz BT-Q1000XT) and ActiGraph GT3X+ accelerometers for eight consecutive days during waking hours. GPS data were recorded at 10-second intervals, whilst accelerometers collected raw triaxial acceleration data at 100 Hz, aggregated into 10-second epochs with activity intensity classified using ActiGraph’s standard count-based thresholds [46]. This enabled us to estimate the time spent in sedentary behaviour, as well as in light, moderate, or vigorous intensity physical activity which was needed as part of identifying children’s AST [45]. Ethical approval was provided by the College of Social Sciences, University of Glasgow (CSS ref: 400140067).

Of the 774 participants (417 girls and 357 boys) who completed the device wearing protocol, approximately 10% had incomplete data (defined as fewer than four valid weekdays of GPS tracking), leaving 695 valid responses. Since the study did not directly ask about travel mode to school, we applied a systematic approach to identify participants likely to have walked to school (see Sect.  2.3), resulting in a final analytical sample of 233 participants for this study.

It is important to note that whilst the original SPACES dataset is representative of Scottish children when appropriate weights are applied, our specific selection criteria for identifying walking trips rendered these population weights inappropriate for this analysis. Our sample therefore represents children who walked to school within the SPACES cohort rather than the broader Scottish population, and no sample weighting was applied in the statistical analyses.

### Variables

#### Sex and socioeconomic status

Each child’s GPS data were linked to Growing Up in Scotland (GUS) Sweep 8 data [47], which provided their sex and home area socioeconomic position, as determined by the 2016 Scottish Index of Multiple Deprivation (SIMD) [48]. The SIMD is a comprehensive area-based index that considers income, employment, health, education, skills and training, housing, geographical access to services, and crime. The index ranks data zones (small area units of ≈ 750 persons) in Scotland from most to least deprived. To ensure sufficient statistical power for analysis, we created a dichotomous variable for deprivation, combining SIMD quintiles 1–3 as “more deprived” (30% of participants) and SIMD quintiles 4–5 as “less deprived” (70% of participants) [47].

#### Locations of homes and schools

Each child’s home and school address were provided as part of the consent process and subsequent data sharing agreement between the participants, their parents, the University of Glasgow and the Scottish Government/GUS. These data were handled securely [49]. The addresses allowed us to generate routing information using our preferred routing engines.

#### Urban/Rural classification

We employed the Scottish government’s binary classification system to categorise children’s school journeys as urban or rural [50]. Rather than using residential address alone, we determined the classification based on the predominant landscape type encountered along each child’s school commute route. This approach accounts for journeys that cross both urban and rural landscapes by using the statistical ‘mode’ to identify the most frequently occurring category along each route.

#### Road networks

The road networks of both Mapbox and the Open Source Routing Machine (OSRM) derive their data from OpenStreetMap (OSM), whilst Google maintains a proprietary network with confidential data sources [51, 52]. The OSM community employs a variety of methods to detect inaccuracies within the dataset, utilising numerous quality assurance tools and a reporting system that enables users to highlight potential errors or regions requiring review by individuals with local expertise. Mapbox also has a dedicated technical and review system in its product to automatically find and separate changes that might be mistakes or harmful actions [53]. In practice, a walking route is obtained by submitting origin and destination coordinates to each engine’s API with the travel mode set to “walking” for Google and Mapbox or “foot” for OSRM. The API returns one or more candidate routes as encoded polyline geometries together with total distance and estimated duration^1^. Google’s routing API publicly documents many of its routing options, however, the precise internal cost function and edge-weighting scheme remain undisclosed. For example, pedestrian-friendly paths such as shortcut paths or complexes are not marked on public maps. Walking directions from all three engines are time-invariant, unlike driving directions which account for real-time traffic conditions.

To facilitate a comparison between these routing engines and the existing distance-based routing, we used the Ordnance Survey MasterMap Integrated Transport Network (ITN, version April 2018) provided by Edina Digimap [54]. Using the participants’ origin and destination points, we computed shortest-distance paths based on Dijkstra’s algorithm [55].

### Obtaining ‘real’ routes for journeys from home to school

Our analysis focused solely on journeys where children walked from home to school, and this choice was driven by several considerations. Walking represents the most common form of active transport for school journeys and aligns with the SPACES study’s focus on children’s physical activity patterns. Unlike vehicular routes, walking allows children greater route choice flexibility, including access to footpaths, park routes, and shortcuts not available to motorised transport.

We found that it was necessary to remove GPS points around children’s homes and school locations due to the phenomenon known as ‘GPS scatter’, in which GPS points can appear dispersed due to interference or weak signals, particularly indoors. Such scatter may suggest longer or more complicated routes than that actually taken. We achieved a cleaner, more representative depiction of the child’s route by removing these scattered points, particularly at the beginning and end of each journey. The finer details of each of these steps are described below:

#### Identifying children who walked to school

To infer which participants walked to school, we applied a systematic 2-step process to the individual-level GPS data. In Step 1, we identified the GPS data recorded between 07:00 and 09:00am. The decision to select only the journey *to* school was based on the observation that many children participate in post-school activities, which often align with their parents’ work schedules [30, 33]. We note that routes to and from school may not be symmetrical; journeys to school may be more direct due to time pressure, whereas return journeys may involve detours to visit friends, shops, or recreational areas. Our analysis therefore captures only one direction of the school commute, and findings may not generalise to homeward journeys.

In Step 2, we applied speed and accelerometer filters to the GPS data to select the journeys that were most likely walked. GPS data from participants whose speed at any point did not exceed 9 km/h was used, as this speed is an indication of fast walking or light jogging [56]. We conducted a sensitivity analysis for upper-speed thresholds, ranging from 5 km/h to 7 km/h and extending to 9 km/h. The 9 km/h threshold was selected because we identified several cases where children moved at this speed whilst registering vigorous physical activity on accelerometers, indicating genuine walking or brisk walking rather than vehicular travel. Using accelerometer data that classified time spent as sedentary, light, moderate, or vigorous, we excluded participants who moved slower than 9 km/h but were also classified as ‘sedentary’, as this combination likely indicated vehicular travel [47].

#### Cleaning GPS tracks at home and at school

The SPACES GPS data frequently indicated a high concentration of points around home locations and within school playgrounds, which reflects the considerable amount of time children spend in these areas during the mornings [57]. However, to accurately capture trip data, it was necessary to exclude movements that occur inside homes and around school entrances.

For this study, we applied a 20-metre buffer zone around each child’s home and excluded all GPS points recorded within this buffer. This buffer size was selected because it encompasses most housing types in Scotland, including tenements and semi-detached houses. This ensures that we captured the actual start of the walking journey rather than movements within or immediately around the home.

To remove accumulated data points from school playgrounds, we identified the first GPS point within the school boundary as the ‘arrival’ point and excluded all subsequent points. We downloaded the school boundaries from the OpenStreetMap database using the QGIS “QuickOSM” function with the key: amenity and value: school for the query. 

**Fig. 1:**
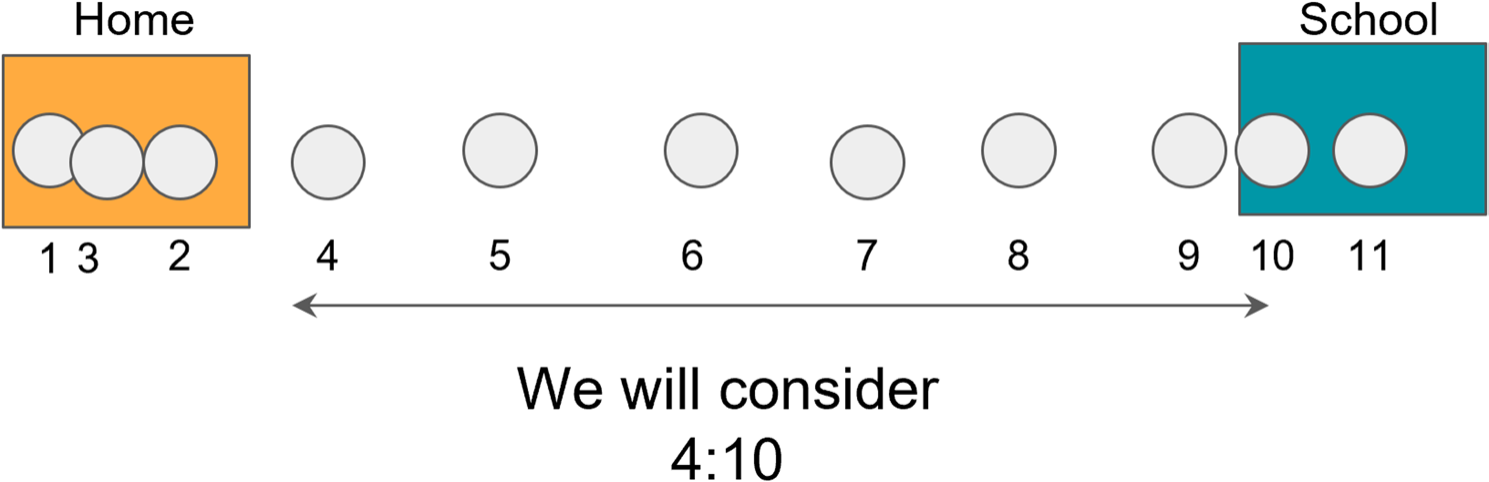
summarises these steps by illustrating a home-to-school journey with eleven GPS points, four to ten of which were recorded for each child for analysis

Figure 1 Illustration of a home-to-school journey with 11 GPS points. GPS points 1 to 3 were within 20 m of the home location, and points 10 to 11 were within 20 m of the school location. Therefore, for the purposes of our analysis, step four marks the beginning of the walk, and step ten denotes the school’s arrival point. From the refined dataset, we systematically identified complete home-to-school journeys during weekdays for all participants.

### Modelling home-to-school routes using API-based navigation engines

To calculate the most direct walking routes from participants’ homes to schools, we employed three routing engines: Google API, Mapbox API, and the Open Source Routing Machine (OSRM). These engines facilitated the extraction of the suggested route data on walkable paths. These data were then exported as GIS polylines. The derived geospatial files included parameters such as longitude, latitude, distance (m), trip duration (minutes), walking speed (up to 4 km/h, the default speed assumed by the routing engines for pedestrians, distinct from the 9 km/h threshold applied to GPS data to identify walking trips), number of turns, and journey stages (i.e., distinct segments of the route between waypoints or direction changes). To align the routes with the GPS data, the addresses of the children’s homes and schools served as input parameters. Routes from the three engines were configured in ‘walking mode’. We used specific R packages designed for each API: mapsapi [42] for accessing the capabilities of the Google API, mapboxapi for Mapbox [41], and osrm for OSRM [51]. Whilst these are different packages, each follows consistent R geospatial data conventions, enabling uniform analytical processing and comparison across all three routing engines.

We buffered the model-generated polylines by 20 m to: (1) reflect the typical width of residential roads in Scottish urban areas; and (2) account for the positional accuracy of consumer-grade GPS receivers, which can vary by local context (e.g. lower accuracy in dense urban canyons) [58]. This is at the conservative end of buffer distances typically used in comparable studies, which have ranged from 25 m [34, 35] to 50 m [35, 37], and 80 m [41].

### Assessing the overlap between modelled and actual routes

A spatial intersection was conducted between the GPS-derived points and each of the buffered modelled routes to determine the degree of overlap between the polygons, quantified as a percentage [35, 37, 38, 59]. We refer to the percentage overlap between the modelled routes and GPS tracks as *overlapping accuracy* (OA). For children with multiple valid walking trips across different days, we retained only those trips that originated from the child’s registered home address, as recorded on the consent form. Trips departing from a different location were excluded, as well as incomplete journeys. This ensured that each participant’s OA was calculated from a consistent and comparable home-to-school route.

**Fig. 2:**
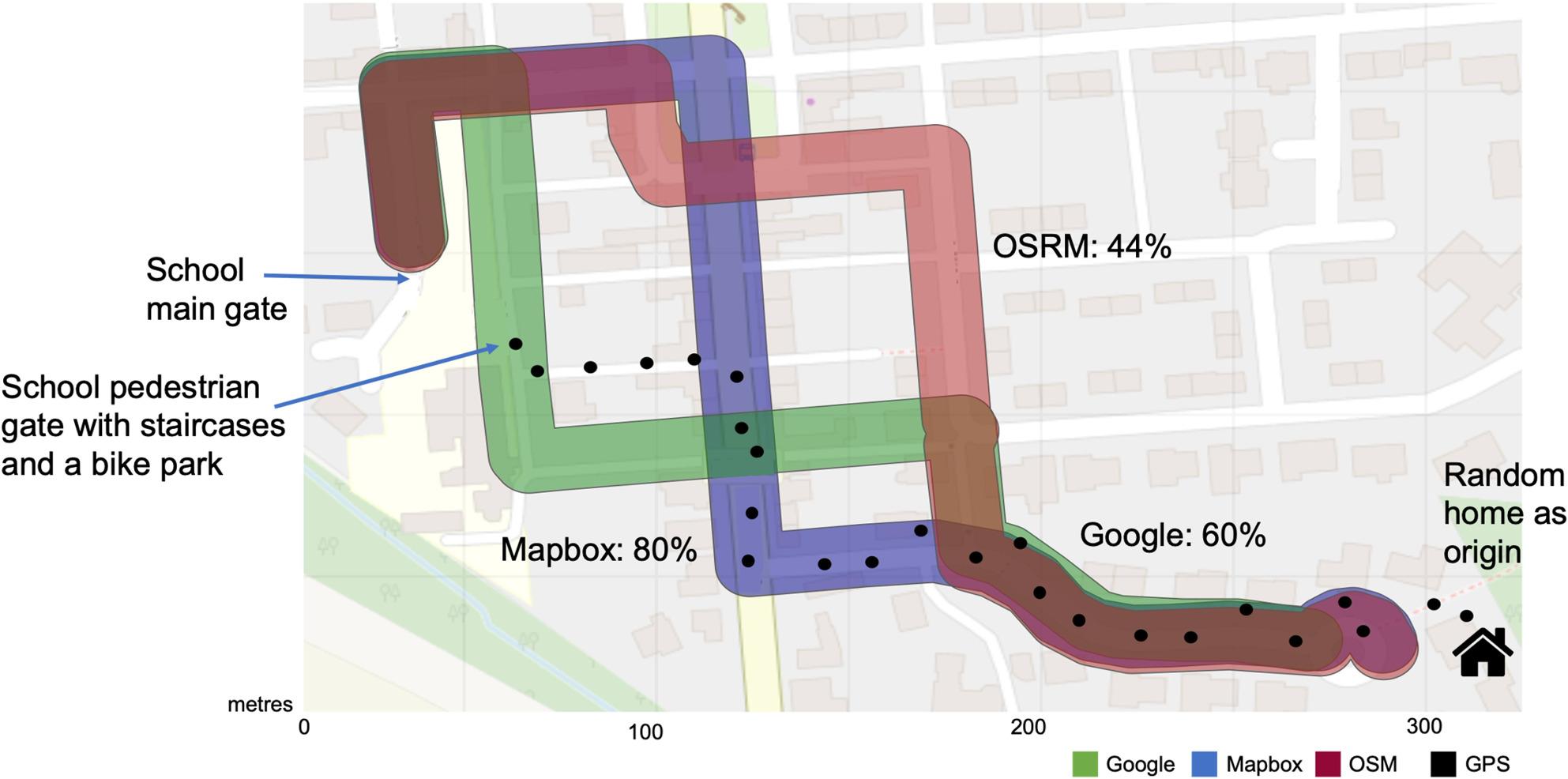
An example of the OA of three routing engines against the GPS trajectory (with black points) in a school journey

OA was used as a measure of similarity between the routes from the routing engine and the GPS trajectories. An OA of 0% means that there is no overlap between the two routes, whereas an OA of 100% denotes a perfect alignment. In Fig. 2, for example, the OSRM and Google engines suggested the child would turn right at the first street, but Mapbox suggested the right turn was made at the next available junction. All three routing engines pointed towards a main gate of the school, yet the child opted for the pedestrian gate. In this example, out of 25 GPS points, the Mapbox routes intersected with 20 points (an 80% match), Google’s routes overlapped with 15 points (a 60% match), and OSRM’s routes aligned with 11 points (a 44% match).

### Statistical analysis

To assess associations between individual or area characteristics and OA, we applied the Wilcoxon Signed-Rank Test [60]. The test was selected because our response variable (OA) showed a left-skewed distribution, characterised by negative skewness, where the mean was less than the median with most observations clustered at higher values and a long left tail. For each of the three methods, we used sex, SIMD, and urban/rural setting as our predictors.

To examine whether route distance influenced routing engine accuracy, we computed Pearson correlation coefficients between GPS-measured walking distance and OA for each engine. The total length of the GPS trajectory is measured by the Euclidean distances between consecutive GPS points of the journey. Since the GPS paths were cleaned in the previous stages, it gives the total distance actually walked as measured by the GPS. We also compared the total route distances returned by each engine against the GPS-derived track distances to assess whether the engines systematically over- or under-estimated travel distance.

### Comparing routing engine performance at an individual level

To assess consistency in routing engine performance across individuals, we calculated pairwise differences in overlap accuracy (OA) between each combination of routing engines (Mapbox vs. Google, Mapbox vs. OSRM, and Google vs. OSRM) for each participant.

We established three categories of individual-level routing consistency based on the magnitude of these OA differences. Participants were classified as having “consistent” routing performance when all pairwise differences were less than 5%, indicating that all three engines performed similarly for that individual’s journey. When two engines showed similar performance (difference < 5%) but the third engine differed substantially (difference ≥ 5%), we classified this as “one discordant” performance. Finally, when all pairwise differences exceeded 5%, indicating that each engine performed distinctly differently, we classified this as “inconsistent” performance.

This 5% threshold was selected to distinguish between minor variations in routing performance (likely due to small route differences) and substantial discrepancies that might indicate fundamentally different route suggestions or varying engine capabilities for specific journey types. A sensitivity analysis testing thresholds of 3%, 5%, 7%, and 10% confirmed that the overall pattern of results was stable across these thresholds (see Supplementary Material).

## Results

### Routing accuracy by demographic groups

**Fig. 3:**
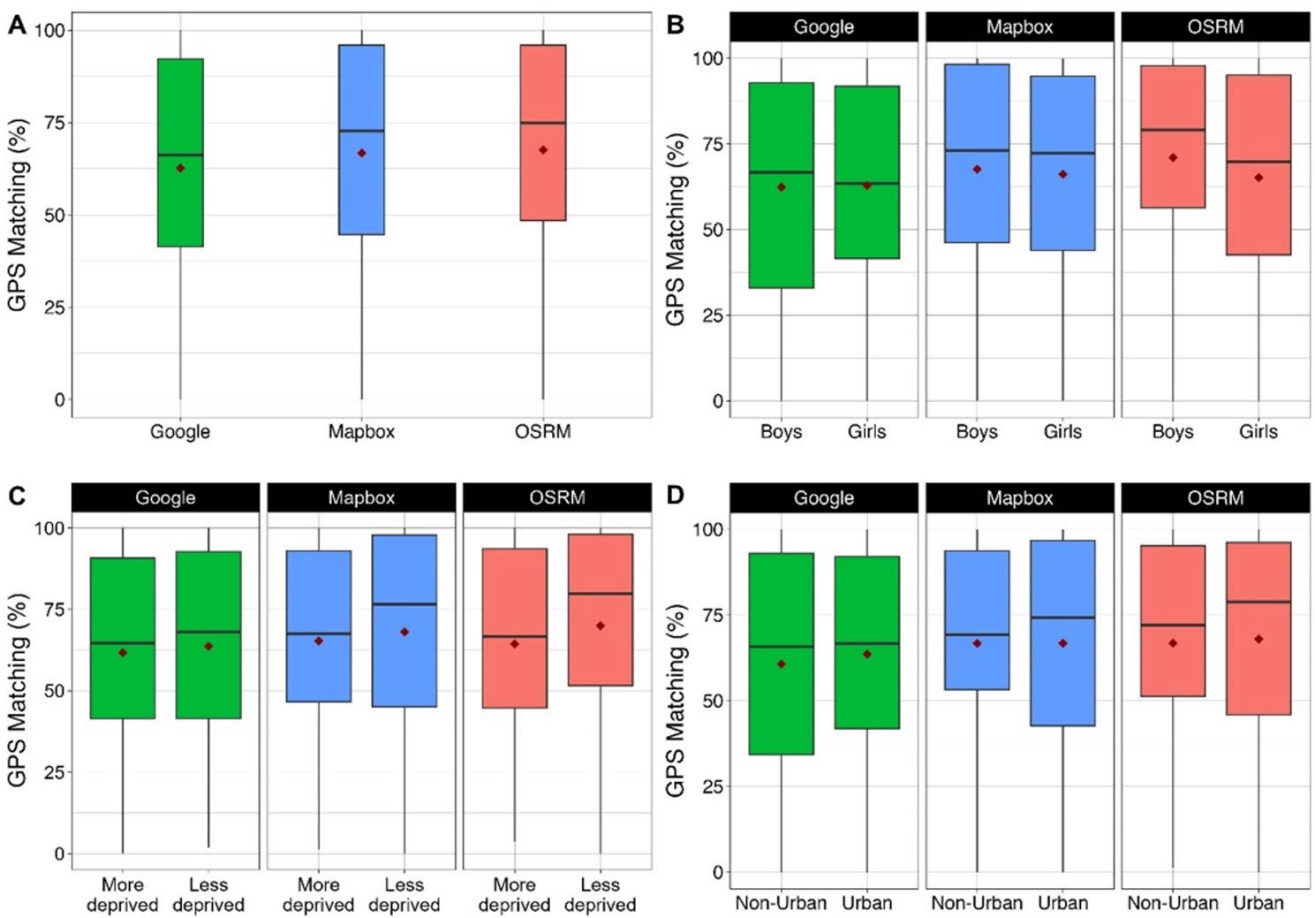
Comparison of the OA of three routing engines in relation to real-world GPS data: overall (**A**), by sex (**B**), by socioeconomic (**C**), and by urban and non-urban (rural) settings (**D**)

Information from the SPACES data was extracted for 233 children identified as having walked to school (42% boys and 58% girls). Among these children, 37% came from more deprived backgrounds, and 63% came from less deprived backgrounds. Regarding their residential context, 67% lived in urban areas, while 33% lived in non-urban areas. The average overlap accuracy (OA) with GPS routes was 67.6% (SD: 30%) for OSRM, 66.8% (SD: 31%) for Mapbox, and 62.6% (SD: 31%) for Google (Fig. 3A). The mean OA for the shortest-distance paths using ITS road networks was 47.3% (SD: 32.4%, *n* = 230), which was lower than for any other routing engine.

When stratified by sex, OSRM showed a 6% higher OA for boys (71%) compared to girls (65%) though this was not statistically significant (*p* = 0.111), while Mapbox (67.6% for boys, 66.1% for girls) and Google (62.4% for boys, 62.8% for girls) displayed negligible sex differences (*p* = 0.442 and *p* = 0.739 respectively) (Fig. 3B; Table 1).

Children from less deprived areas had slightly higher OA across all routing engines (OSRM: 69.9% vs. 64.9%; Mapbox: 65% vs. 63%; Google: 63% vs. 61%) (Fig. 3C; Table 1). However, these differences were also not statistically significant (OSRM: W = 7121, *p* = 0.106; Google: W = 6652, *p* = 0.505; Mapbox: W = 6886, *p* = 0.254).

OAs were also similar in urban and rural areas: OSRM showed 68% in urban areas vs. 66.8% in rural; Mapbox had 66.8% vs. 66.7%; and Google 63.6% vs. 60.7% (Fig. 3D; Table 1). These differences were not statistically significant across all platforms (OSRM: W = 5750, *p* = 0.596; Google: W = 5762, *p* = 0.614; Mapbox: W = 5824, *p* = 0.706). Taken together, overlap accuracy did not vary systematically by sex, area-level deprivation, or urban/rural classification, suggesting that routing engine performance in this Scottish context was largely independent of these area-level and demographic characteristics.

**Table 1:** Wilcoxon test results comparing routing overlaps by sex, deprivation, and urban/rural location across OSRM, Google, and Mapbox

Variable	Group 1	Group 2	Routing	W	*p*-value
Sex	Female (*n* = 136)	Male (*n* = 97)	OSRM	5789	0.111
			Google	6427	0.739
			Mapbox	6206	0.442
SIMD	Less deprived (*n* = 147)	More deprived (*n* = 86)	OSRM	7121	0.106
			Google	6652	0.505
			Mapbox	6886	0.254
Urban	Urban (*n* = 156)	Non-urban (*n* = 77)	OSRM	5750	0.596
			Google	5762	0.614
			Mapbox	5824	0.706

### Overlap accuracy and distance

From the participants journey to school the mean GPS-measured walking distance was around 0.79 km (SD: 0.51). All three routing engines slightly overestimated travel distance relative to GPS-measured tracks (Table 2). Google overestimated by a mean of 42 m, Mapbox by 30 m, and OSRM by 33 m.

A statistically significant but weak negative correlation was found between GPS distance and OA for OSRM (*r* = − 0.222, *p* < 0.001), Mapbox (*r* = − 0.202, *p* = 0.002), and Google (*r* = − 0.134, *p* = 0.041, indicating that longer routes were associated with slightly lower overlap accuracy, though distance explained only a small proportion of the variance in OA. No significant negative correlation was observed for the OS MasterMap ITN baseline (*r* = − 0.078, *p* = 0.238).

**Table 2:** Distance estimation by routing source compared with GPS-measured tracks (*n* = 233)

Source	Mean distance (km)	Mean diff. vs. GPS (m)
GPS (observed)	0.76	
Google	0.83	75
Mapbox	0.82	66
OSRM	0.82	69
ITN Road network	0.95	207

### Performance of routing engines for each individual

We observed substantial variability in the OA of routing engines for individual participants (Table 3). Of the 233 participants, only 78 (33.4%) received similar route suggestions from all three engines. Most participants (155, or 66.6%) had some degree of disagreement between engines.

Nearly half of all participants (115, 49.4%) had one engine which produced a substantially different route compared to the other two engines. Additionally, 40 participants (17.2%) experienced complete disagreement, with all three engines suggesting different pathways for their school journey (see visual examples in the *Supplement file*).

**Table 3:** The consistency outcome of routing engines by individuals

Category	Frequency (%)
Consistent	78 (33.4)
One discordant	115 (49.4)
Inconsistent	40 (17.2)
Total	233 (100)

## Discussion

The main objective of this study was to compare the accuracy of various routing engines in generating home-to-school routes against ‘real’ routes measured using GPS trajectories of 233 Scottish children aged 10–11 years. We compared the actual walked routes with those suggested by three different routing products: OSRM, Mapbox, and Google. We also compared performance across area-level socioeconomic status, for urban and rural settings, and for boys and girls. We further investigated how engine performance varied within individual.

The measure of agreement was overlap accuracy (OA) and, overall, OSRM showed the highest average OA at 67%, with Mapbox at 66% and Google at 63%. On average, approximately two-thirds of the route to school predicted by these products overlapped with the participants’ ‘real’ route. This was significantly larger than the equivalent measure computed on the OS MasterMap ITN network (47%). These levels of agreement are comparable to those reported in related studies. Dalton et al. [37] found moderate to substantial agreement with GPS-recorded trips, with walking trips accounting for 59% overlap, and Klein et al. [61] reported that differences between modelled and observed routes were for walking trips (28%) than for car trips (16%) among older adults (aged 67–94) in Luxemburg the shortest routes often intersecting near amenities and green spaces.

However, we observed no significant differences in OA for children with different area-based socioeconomic status, sex or urban/rural location, across the three engines. This implies that use of these routing engines to estimate AST route did not introduce bias or systematic variation related to participant characteristics. This does not, of course, mean that real behavioural differences between groups, perhaps founded in safety concerns or aesthetic preferences [19, 20, 32], are not present in real life.

Beyond demographic and area-level characteristics, we also examined whether the physical distance between home and school influenced routing accuracy. We found a significant negative Pearson correlation between route distance and OA for the three routing engines (r ranging from − 0.16 to − 0.23, all *p* < 0.05), indicating that longer routes were associated with lower overlap accuracy, though the effect was weak. This is broadly consistent with earlier findings: Harrison et al. [35] found that every additional kilometre between home and school increased the odds of greater length discrepancy between GIS-modelled and GPS-measured routes among 13 to14 year-olds, whilst Dalton et al. [37] reported a statistically significant but low correlation (*r* = − 0.309) between distance difference and percentage overlap among adult commuters.

This is broadly consistent with earlier findings. Dessing et al. [33] found that Dutch children’s actual walking routes were on average 5.6% longer than the shortest network path, with a median overlap of 64% at a 25 m buffer, suggesting that children do not simply follow the shortest available route. Our findings extend this evidence by showing that the distance-accuracy relationship holds not only for shortest-path models but also for modern routing engine predictions, and that longer journeys provide more opportunities for route divergence. No significant correlation was observed for the OS MasterMap ITN baseline (*r* = − 0.08, *p* = 0.238), likely because the road-only network lacks pedestrian infrastructure. We used a road-only baseline because pedestrian path networks are not reliably or consistently defined, even by the UK national mapping agency, resulting in consistently low accuracy regardless of route length. Origins and destinations were set to the nearest node on the ITN network; however, three of the original 233 participants could not be routed because their snapped locations fell on disconnected components of the road graph. Thus, ITN has results for 230 out of 233 participants. All three engines slightly overestimated travel distance relative to GPS tracks (mean difference: 30–42 m), which is consistent with children not always following the shortest available path.

However, individual participants did often experience inconsistent routing across engines. Only 78 out of 233 participants showed consistent routing across all the engines. This variation in routing engine performance was independent of the individual and contextual factors examined in Sect.  3.1, suggesting that route-specific characteristics may influence engine accuracy. The substantial proportion of disagreement between routing engines suggests they use different road, path and trail databases and/or that their routing algorithms are more distinct than might be expected. Additionally, despite all engines prioritising the shortest path and shortest time, each has a unique decision hierarchy (e.g., number of turns, road prioritisation, and determining school entrance by its front or rear gate). These differences become explicit in complex built environments, which typically have dense street networks, and irregular block shapes. In such areas, high street connectivity and ambiguous entry points, such as informal pathways and multiple entry gates, may lead to different routing options.

The observed OA was substantially higher than that found in earlier AST studies. Harrison et al. [35] and Mizen et al. [34], reported average OAs of 56% and 18%, respectively. In related research, an average OA of 54.7% was observed [40]; however, this involved a participatory mapping method that required children to map their routes manually through an online web page, serving as the reference trajectory. The relative high OA in our study perhaps suggests improvement in route identification technologies, but we cannot rule out that it might also reflect improvements in the underlying databases and algorithms used by these engines. Either way, it seems that contemporary routing engines increasingly reflect real-world pedestrian movements.

This increase in OA in comparison to previous studies may be partly attributed to the use of advanced databases and APIs that are fine-tuned for pedestrian needs, which were not used in earlier research [33–35, 39]. Moreover, the application of a ‘snapping’ technique that corrects for GPS inaccuracies by aligning recorded paths to the nearest roads has contributed to this enhanced accuracy, even with a more precise 20 m buffer, as opposed to the wider buffers employed in previous studies [35, 40]. This indicates a significant step forward in the reliability of routing engines for practical applications, especially compared to older GIS-based methodologies and manual participatory mapping methods. The improved average OA in comparison to past studies does not negate the value of GPS-based research. Our findings indicate that approximately 33% of routes do not align with the directions suggested by routing engines. The variability in accuracy can be attributed to numerous factors, such as the characteristics of the built environment and individual travel preferences. Routes passing through high-traffic zones and avoiding residential areas often result in lower OAs [33–35]. In contrast, routes perceived as safer by pedestrians, typically main roads and commercial areas, tend to have higher OAs [40, 62].

The study had some limitations. First, although we managed to align them with the nearest road network as accurately as possible, some of the GPS footprints did not overlap perfectly with the routes generated by the routing algorithms. Hence that left a few percent of uncertainty in the spatial alignment between the observed and modelled routes. Second, the study focused solely on children walking to school and did not consider other modes of transportation, such as cycling, scooters, or motorised transport, which may have different implications for routing accuracy [30, 63]. Third, our sample under-represents children from more deprived areas: SIMD quintiles 1–3 account for 60% of the Scottish population but only 30% of our analytical sample, a pattern typical of GPS-based studies that require sustained participant compliance. Fourth, there is a temporal misalignment between the GPS data collection during 2015–2016, and the routing engine queries requested in 2024. Whilst the physical road network in these Scottish residential areas has remained largely stable, pedestrian path data and routing algorithms have likely been updated over this period. We were unable to obtain historical routing engine outputs, as these APIs do not provide archival access. Fifth, the 20 m buffer used for overlap assessment introduces some spatial imprecision; in dense urban areas, a 20 m buffer could encompass parallel streets or paths, potentially inflating OA. Map-matching GPS points directly to the network would provide greater precision and represents a methodological improvement for future research. Sixth, approximately two-thirds of participants showed some degree of disagreement between engines, suggesting that choice of routing engine could materially affect study conclusions; this inconsistency across engines should be considered when interpreting routing-based AST studies. Seventh, our findings pertain specifically to 10- to 11-year-old children who walked to school within the SPACES cohort in Scotland, and generalisability to other age groups, travel modes, or geographical contexts should not be assumed without further validation. We also lacked information on whether children walked alone, with peers, or with an adult, which would likely influence route choice. Finally, whilst our methodology demonstrates broad applicability given the global availability of these routing engines, local adaptations may be necessary when applying this approach in different geographical contexts. For instance, our 20-metre buffer was specifically chosen to reflect typical Scottish residential road widths, but countries with wider road infrastructure would require adjusted buffer sizes to maintain methodological validity. Similarly, commuting routes and travel behaviours can vary across regions, urban settings, and cultural contexts. However, our demonstration that routing engine accuracy is independent of individual and contextual factors suggests this approach offers a robust foundation for international studies of children’s mobility patterns, provided that local infrastructure characteristics are appropriately incorporated into the analytical framework.

## Conclusion

This study assessed the OA of GPS-tracked routes for school commutes by 10–11-year-old children in Scotland (data collected 2015–2016) as estimated by Google Maps, Mapbox, and OSRM in 2024. The OA for these engines ranged from 63 to 67%, showing no substantial differences between them in terms of performance. The OA of the routing engines did not vary by respondents’ individual or contextual characteristics. The observed average OA represents a good performance, particularly compared to that observed in older studies. However, more than a quarter of the participants were provided with substantially different routes by different routing engines. Overall, contemporary routing engines can complement GPS in children’s mobility research and practice, offering an empirical reference point for future active school travel studies.

## Supplementary information

Supplementary material 1.

## Data Availability

Studying Physical Activity in Scotland (SPACES) data used in this study are confidential and cannot be made openly available. Summary data may be available upon reasonable request subject to approval by the data custodian. Growing Up in Scotland (GUS) Cohort 1 Data can be accessed via the UK Data Service through Secure or Special Licence DOI: [http://doi.org/10.5255/UKDA-Series-200020](http:/doi.org/10.5255/UKDA-Series-200020) . The code for which the data were analysed can be provided upon request.

## Footnotes

1 OSRM is fully open-source and pre-processes the OSM graph using Contraction Hierarchies, a speed-up technique that enables near-instantaneous shortest-path queries; its “foot” profile restricts traversal to edges tagged as walkable (e.g. footway, path, residential) and applies a default walking speed of approximately 5 km/h with penalties for surface quality and gradient. Mapbox’s Directions API is built on the Valhalla routing engine, which organises the OSM network into a tiled, hierarchical graph and applies an A* search; its pedestrian costing model favours footpaths and living streets whilst penalising high-traffic roads, and Mapbox applies additional proprietary quality filters to the underlying OSM data before routing. Google’s Routes API computes pedestrian paths over its own proprietary road network; the API documentation describes configurable route modifiers (e.g. avoidance of indoor paths and stairs) and notes that walking routes may include footpaths and pavements not available to vehicles (https://developers.google.com/maps/documentation/routes).
