# Supplementary material for: Assessing the accuracy of routing engines in replicating children’s walking routes to school: a comparative study of google, mapbox, and OSRM
Source: Int J Health Geogr. 2026 May 19;25:38. doi: 10.1186/s12942-026-00473-7 (PMC13397760; doi:10.1186/s12942-026-00473-7)
Supplement: Supplementary file 1 — Supplementary material 1. [file 12942_2026_473_MOESM1_ESM.docx]

# Appendix

## A1. Individual OA between routing algorithms


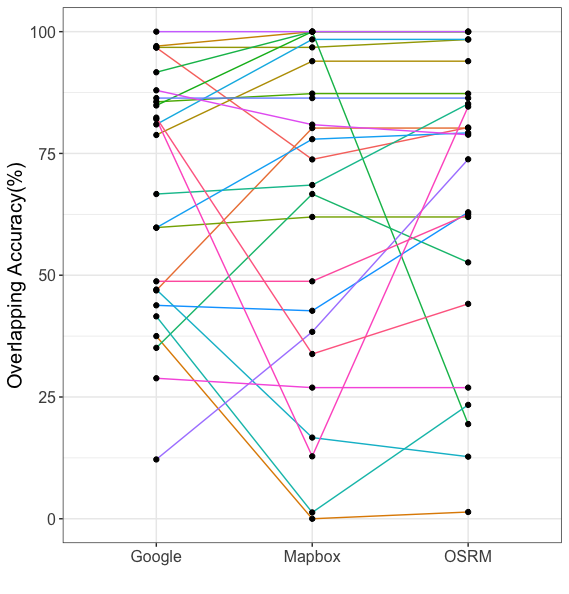


Figure S1 Individuals’ OA in three different

This is a sample of 25 participants' OA from three routing engines: Google, Mapbox, and OSRM. Each line demonstrates an individual's OA across the three approaches.
